# Individual and Combined Cytotoxic Effects of Co-Occurring Deoxynivalenol Family Mycotoxins on Human Gastric Epithelial Cells

**DOI:** 10.3390/toxins9030096

**Published:** 2017-03-09

**Authors:** Yunxia Yang, Song Yu, Yanglan Tan, Na Liu, Aibo Wu

**Affiliations:** SIBS-UGENT-SJTU Joint Laboratory of Mycotoxin Research, Key Laboratory of Food Safety Research, Institute for Nutritional Sciences, Shanghai Institutes for Biological Sciences, Chinese Academy of Sciences, University of Chinese Academy of Sciences, Shanghai 200000, China; yxyang@sibs.ac.cn (Y.Y.); syu@sibs.a.cn (S.Y.); yltan@sibs.ac.cn (Y.T.); liuna@sibs.ac.cn (N.L.)

**Keywords:** deoxynivalenol, mycotoxins, human gastric epithelial cell, cytotoxicity, combination index

## Abstract

Mycotoxin contamination is a significant health concern for human beings, but health risk assessments are usually based on one single mycotoxin, which might neglect the additive or competitive interactions between co-occurring mycotoxins. In this study, we assessed the individual or combined toxicological effects to multiple deoxynivalenol-family mycotoxins, namely deoxynivalenol (DON), Nivalenol (NIV), and their acetyl derivatives of 3-acetyldeoxynivalenol (3-ADON), 15-acetyldeoxynivalenol (15-ADON), deoxynivalenol-3-glucoside (D3G), and Fusarenon-X (FX) based on the human gastric epithelial (GES-1) cells. GES-1 cells were treated at different concentrations over 24 h and cell viability was measured by a cell counting kit (CCK8). The results show that D3G has no toxicity and 3-ADON is less potent in reducing cell viability compared to DON, whereas 15-ADON and FX appear to be slightly less potent than their parent compounds of DON and NIV on GES-1 cells. In general, the toxic ability of individual mycotoxins was shown as 3-ADON << 15-ADON < DON < FX < NIV, in an increasing order. All mixtures caused a dose-dependent decline of cell viability and the interactions analysis of binary combinations were assessed using the combination index (CI)-isobologram method. For the interaction types of mycotoxins mixtures, the synergistic cytotoxicity of DON + 15-ADON, DON + NIV, and DON + FX at low and/or moderate inhibitory concentration levels (IC10–IC70, IC10–IC80, and IC10–IC40, respectively) were observed. FX + NIV resulted in almost completely synergistic cytotoxicity, whereas 15-ADON + NIV and 15-ADON + FX presented almost entirely antagonistic cytotoxicity on the GES-1 cell model. These results suggest that the simultaneous presence of low-dose type B trichothecenes in dietary food may be more or less toxic than the prediction based on individual mycotoxins.

## 1. Introduction

Trichothecenes are secondary metabolites produced by fungi belonging to the *Fusarium* genus that commonly invade various crops and cereals with high contamination levels and occurrence frequency worldwide [1]. They can also be found in animal-derived food due to contaminated feed and silage, such as meat, eggs, and milk and milk-based products [2]. Trichothecenes mainly affect the immune system and gastrointestinal tract and cause vomiting, anorexia, gastroenteritis, and immunological dysfunction, with subsequent losses of body weight gain, which presents threats to human health and animal production and creates economic costs [1,3,4].

Deoxynivalenol (DON) is one of the most prevalent mycotoxins in the trichothecene family and cannot be completely eliminated during food processing operations [5]. Hence, DON contaminants in crops and their final processed food products are of worldwide concern. The FDA advisory level for DON or a provisional maximum tolerable daily intake (PMTDI) is 1000 µg/kg (1 ppm) for wheat products [6]. Of note, trichothecenes often exist in natural circumstance by the modified forms that originated from their parent compounds. In plants, they have their own detoxification systems as a form of defense against exogenous harmful substances. Specifically, plants can alter the chemical structures of such mycotoxins by enzyme-catalyzed reactions, called detoxification processes [7]. The modified forms are typically called “masked” mycotoxins [8]. The appearances of masked mycotoxins bring some changes, including chromatographic patterns, epitope conformation, or molecular polarity, however, our confirmed conventional analytical methods cannot detect these masked mycotoxins, which still have no safety limit standards [6,9,10,11]. The acetylated and glycosylated derivatives of parent DON, including 3-acetyldeoxynivalenol (3-ADON), 15-acetyldeoxynivalenol (15-ADON), and deoxynivalenol-3-glucoside (D3G), could also be detected in wheat and corn [6,9,12]. It is worth mentioning that the currently established safety limit regulations were mainly derived from toxicological data, which only take into account single-mycotoxin exposure without considering the combined mycotoxins effects [13]. Obviously, the individual mycotoxins’ toxicities cannot represent the real toxicity of mycotoxin co-occurrence and ignores the interactions among mycotoxins, i.e., additive, synergistic, or antagonistic toxic effects. In addition, one or more type B trichothecene co-occurrences in the natural world were also reported by several surveys, and mainly produced by *F. graminearum* and *F. sambucinum*, and also consist of *Myrothecium*, *Stachybotris*, *Microcyclospora*, *Trichothecium*, *Trichoderma*, etc. [14,15]. The data on combined toxic effects of mycotoxins are limited; therefore, the health risk of combined toxicity from exposure of multiple mycotoxins have been poorly understood so far. Owing to the natural occurrence of type B trichothecenes together in grains and cereals is well-known [16,17,18]; it is of the utmost significance to assess the toxicological effects of mycotoxin interactions to provide better health risk evaluation of such mycotoxin contaminations.

The gastrointestinal epithelium is the first target exposed to mycotoxins, even under higher doses compared with other tissues [19,20,21]. However, studies on the interaction effect of mycotoxin mixtures on the gastrointestinal tract are rare. Herein, we chose the human normal cell lines—human gastric epithelial cells (GES-1)—as an in vitro cell model to study the toxic effects of individual trichothecene family members and their mixtures to assess individual and combined mycotoxin toxicological effects. Of note, we use the studied parameters—cell viability—as the main endpoint in our experiments.

## 2. Results

### 2.1. Individual Cytotoxicity of Type B Trichothecenes

**Figure 1:**
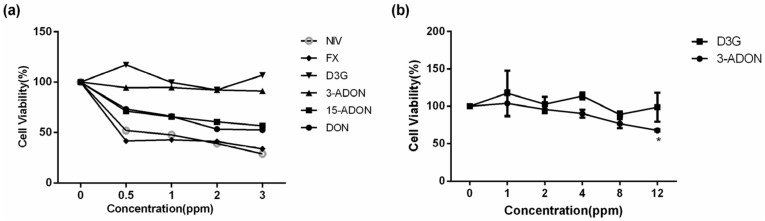
Comparative toxicity of individual type B mycotoxins in GES-1 cells. (**a**) GES-1 cells were exposed to serial dilutions of toxins (DON, 15-ADON, 3-ADON, D3G, FX and NIV) alone for 24 h; (**b**) The cell viability of GES-1 cells treated with a higher dose range of D3G and 3-ADON for 24 h. Data represent means ± SD of three independent experiments; * means *p* < 0.05 compared to the control group.

Cell viability of individual type B trichothecenes was firstly tested on GES-1 cells. The established dose-response curves indicated that the four fusarial mycotoxins (DON, 15-ADON, FX, NIV) induced a marked decrease of cell viability in a concentration-dependent manner (Figure 1a). In contrast, neither D3G nor 3-ADON decreased any cell viability within all of the concentration ranges, even up to the highest concentration of 2 ppm in the initial experiment. The cell viability values of D3G and 3-ADON were also obtained from 1–12 ppm on GES-1 cells (Figure 1b). In addition, an even higher concentration (12 ppm) of D3G did not significantly reduce the cell viability. However, there was a tendency where 12 ppm 3-ADON reduced the cell viability. The results show that D3G shows no toxicity to GES-1 cells and 3-ADON reduced the cell viability to 70% compared to the control when the concentration reached 12 ppm, which indicated that 3-ADON had roughly a ten-fold lesser potency. In consideration of these results, we used DON, 15-ADON, FX, and NIV in the test of combined mycotoxins. Taken together, the effective toxicity of individual mycotoxins diminishing cell viability in increasing order is: D3G < 3-ADON << 15-ADON < DON < FX < NIV.

### 2.2. Combined Toxicity of DON, 15-ADON, FX, and NIV on GES-1 Cells

**Figure 2:**
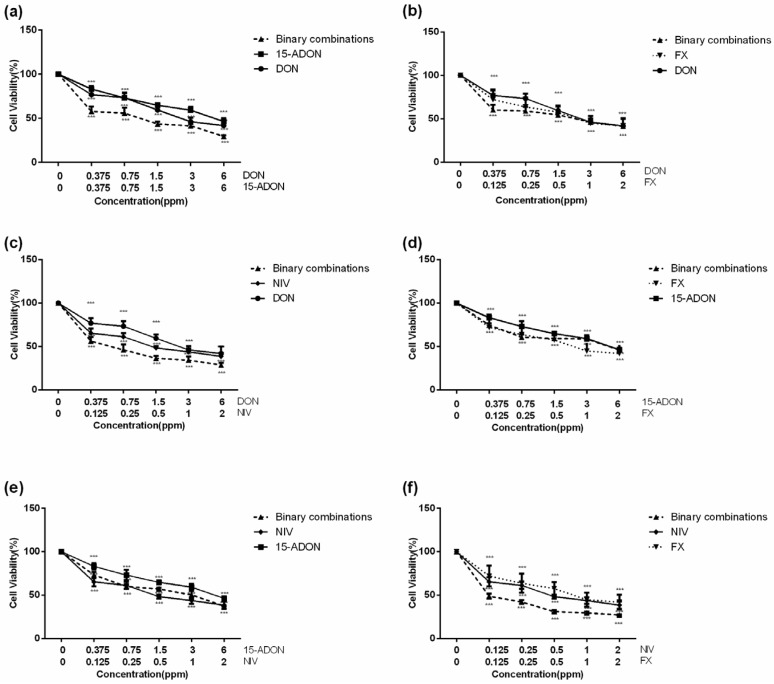
Cytotoxicity of single mycotoxins DON, 15-ADON, NIV, or FX and their binary mixtures in proliferating GES-1 cells. (**a**–**f**) GES-1 cells were treated with serial dilutions of toxins alone (DON, 15-ADON; DON, FX; DON, NIV; 15-ADON, FX; 15-ADON, NIV and FX, NIV) or in combinations (DON+15-ADON, DON+FX, DON+NIV, 15-ADON+FX, 15-ADON+NIV and FX+NIV) for 24 h and cell viability was assessed by the CCK8 assay. Data are the mean ± SD of three independent experiments; *** means *p* < 0.001, significantly different from the control group.

The cell viability of the dose-effect relationships curves for the toxicity of the tested mycotoxin mixtures is presented in Figure 2. In trichothecenes combinations, the concentrations are presented on the abscissa: DON(0.375–6 ppm), 15-ADON (0.375–6 ppm), NIV (0.125–2 ppm), and FX (0.125–2 ppm). The results showed that the cell viability values of all binary combinations were decreased in a dose-dependent manner and approximately declined to 30% with a maximum amount using the high dose of mixtures compared to the control. In particular, binary combinations of FX + NIV exhibited the most potency in decreasing the cell viability compared with other mycotoxin mixtures, while 15-ADON + FX had the least effect. The cell viability declined to approximately 50% with the high concentration (6 ppm + 2 ppm) of 15-ADON + FX.

The results of the dose-effect relationship parameters derive from in vitro cell viability studies are listed in Table 1. The correlation coefficients (*r*) were obtained from the median-effect plots. This revealed good linear correlation coefficients with *r* > 0.95, which means it is eligible and acceptable for further data analysis using the median-effect equation. The results showed IC50 values ranged from 0.18 to 4.79 ppm in binary combinations.

### 2.3. The Different Interaction Effects of Type B Trichothecenes

The interaction effect of mycotoxin mixtures mainly include three main different effects: synergistic, additive, and antagonistic. The types of interactions were decided by the CI values that were calculated by the isobologram method. The CI and DRI values at different cytotoxicity levels (IC10–IC90), i.e., *fa*, are shown in Table 2. The analyzed results show the combinations of DON and its acetylated derivative, 15-ADON mainly resulted in synergistic cytotoxicity at low and moderate *fa* values (cell inhibition levels from 10% to 70% = IC10–IC70) on GES-1 cells. FX+NIV mixtures resulted in almost complete synergistic cytotoxicity (IC10–IC90) on GES-1. In addition, a synergistic effect was also found with exposure to DON + NIV in combination at IC10–IC80, whereas DON + FX combinations resulted in a synergistic effect at low cell inhibition levels (IC10–IC40) and an antagonism effect occurred at a higher cytotoxicity level. For 15-ADON + NIV, a completely antagonistic effect (IC10–IC90) was observed in GES-1 cells. Consistent with 15-ADON + NIV, 15-ADON + FX also resulted in almost entirely antagonistic cytotoxicity at IC20–IC90. The dose reduction index (DRI) represents a multiple of the dose reduction of the dose of tested toxin combinations compared with that of each individual toxin at the same inhibition rate. Obviously, in numerical value, the CI values are opposite to the two DRI values or at least one of them. The results are also presented in Table 2. When synergy occurs in the effects of the mixtures, the values of dose reduction indices (DRI) were favorable for dose-reduction.

## 3. Discussion

The trichothecene mycotoxin co-exposures commonly occur in food and feed, at various extents, all over the world [18], but our food safety regulations were formulated only based on the toxicity data of individual toxins. A blank still exists in our health safety regulations on the toxicity limit standards.

In addition, the outbreak of gastroenteritis throughout history is closely related to the ingestion of trichothecene contaminations [1]. Many studies on the intestinal epithelium cytotoxicity used the Caco-2, IPEC cell model, ignoring that the stomach is the first target to contact the high concentration of toxin. Hence, it is necessary to study the interaction between toxin combinations and our chosen cell model—human gastric epithelial (GES-1) cells.

Initially, to study the types of binary toxin combinations, we need to confirm the individual toxicity levels of DON, NIV, and their derivatives according to the experimental design. These cell viability results suggested that GES-1 cell rapidly responds to DON, NIV, and their acetylated derivatives 15-ADON, and FX at a concentration of 0.5 ppm, whereas 3-ADON and D3G had no immediate cytotoxicity up to a concentration of 2 ppm, which showed significantly less toxicity than other mycotoxins. In our study, the results of the comparable experiment with D3G and 3ADON indicated that D3G had no effect on decreasing GES-1 cell viability, 3-ADON was less potent than that of DON, whereas the toxicity ability of 15-ADON and FX were slightly below the toxic effect of their parent compounds, namely DON and NIV. In contrast to other reports, there is a slight accordance in the individual toxicity potency for such trichothecenes. For example, Alassane-Kpembi et al. also observed the cytotoxicity of individual type B trichothecenes using proliferating intestinal epithelial cell Caco-2 and confirmed the toxicity potency: 3-ADON < 15-ADON ≈ DON < NIV ≪ FX [22]. With respect to the 3-ADON mycotoxin, the same author reported that 3-ADON exhibits less toxicity than DON to pig intestine cell (IPEC-1) [23]. However, it is worth mentioning that our current results suggested D3G had no toxic effect or biological activity to human gastric epithelial cells. This was in accordance with the recent study of Pierron et al. [24] regarding the modest difference in cytotoxic ability of individual type B trichothecenes. There are some differences that may explain this: one, the selected in vitro cell models; two, the chosen evaluation methods; three, the toxin exposure time; and, four, errors from the experimenters.

Obviously, cytotoxic effects of individual type B trichothecenes have been extensively studied. Nevertheless, with regard to studies on combined toxic effects of mycotoxin mixtures are rare. There is a relatively good accordance between our results and that of others. In our results of mycotoxin mixture interaction, the synergistic cytotoxicity of DON + 15-ADON, DON + NIV, and DON + FX at low and/or moderate cell inhibition levels (IC10–IC70, IC10–IC80, IC10–IC40, respectively) were investigated. Two published research reports with regard to the type B trichothecene combinations by one author showed that DON and its acetylated derivatives 3-ADON and/or 15-ADON, in combination, mainly resulted in a synergistic effect, especially at low *fa* values (*fa*—cell inhibitory percentage from 10% to 30% i.e., IC10–IC30) on human Caco-2 cells, whereas the other papers reported the interaction type between DON and 3-ADON was an antagonistic effect at low *fa* values (IC10–IC30) on porcine IPEC-1 [20]. The studies about DON and NIV mixtures were the most numerous and the above reports showed that a synergistic effect was observed at IC10–IC50 on Caco-2 cells and at IC10–IC90 on IPEC-1 cells [20,22]. Therefore, from all of the studies, a synergistic toxicity effect was often observed at low cell inhibition levels. The results of our study showed that FX+NIV resulted in almost complete synergistic cytotoxicity, whereas 15-ADON + NIV (IC10–IC90) and 15ADON + FX (IC20–IC90) resulted in almost entirely antagonistic cytotoxicity on GES-1 cell model. To date, little data about the effects of 15-ADON and FX or NIV in combination in in vitro studies has been reported. However, in the natural environment, the co-occurrence of 15-ADON and FX or NIV are also found in the tested sample [1]. From the perspective of the synergistic effect or additive effects (data from others reported) of FX and NIV [20], the antagonistic effect of 15-ADON and FX or NIV was not incompatible. This is a new viewpoint of considering a synergy amongst type B trichothecene mixtures at low concentration cell inhibition levels.

## 4. Conclusions

The combinations of DON + 15-ADON, DON + FX, DON + NIV, and NIV + FX led to synergistic effects at low and/or moderate and/or high cell inhibition level, whereas 15-ADON and FX or NIV resulted in antagonistic cytotoxicity. With a general investigation of the trichothecene mixtures on GES-1 cells, the interactions led to additive or synergistic effects, which might be been considered in the mycotoxin regulation. In particular, a synergistic toxicity effect at low cell inhibition levels should be added to the formulation of regulation.

## 5. Materials and Methods

### 5.1. Toxins and Chemicals

Deoxynivalenol (DON), 3-acetyldeoxynivalenol (3-ADON), 15-acetyldeoxynivalenol (15-ADON), Fusarenon-X (FX), Nivalenol (NIV), and deoxynivalenol-3-glucoside (D3G) were purchased from Sigma (St. Louis, MO, USA). Dulbecco’s Modified Eagle’s Medium (DMEM) culture medium, Fetal Bovine Serum and penicillin/streptomycin were purchased from Invitrogen (Carlsbad, CA, USA). Cell survival assay was performed using the cell counting kit-8 (DOJINDO, Kumamoto, Japan) according to the manufacturer’s instructions.

### 5.2. Cell Culture

Human gastric epithelial cells (GES-1) are normal human gastric epithelial cells which were purchased from Beijing Beina Chuanglian Biotechnology Institute. The GES-1 cells were cultured in Dulbecco’s Modified Eagle’s Medium (DMEM) culture medium added to 10% FBS and penicillin/streptomycin. After plating when cells reached 80% confluence in a 10 cm dish, the cells were harvested to conduct the cytotoxicity assay.

### 5.3. Experiment Design

To evaluate the individual and combined cytotoxicity of DON, NIV, FX, and their acetyl derivatives, the GES-1 cells were incubated for 24 h at 37 °C in complete DMEM media. Firstly, the single mycotoxin cytotoxicity assay was conducted to determine the toxic effect to obtain a roughly similar toxicity for single mycotoxins. Combination assays were taken by using the following mixtures: DON + 15-ADON, DON + NIV, DON + FX, 15-ADON + FX, 15-ADON + NIV, and FX + NIV, with exposure times of 24 h with the combined toxin ratios (1:1, 3:1, 3:1, 3:1, 3:1, 1:1) based on the ratio of its individual EC50 value on the basis of the principle of attaining a relatively similar toxicity for each tested toxin [23,25]. Toxin solutions were diluted in complete media as the working solutions, and the tested concentrations in each group ranged from 0.125 ppm to 6 ppm. Cells were treated with five dilutions (serial dilution factor = 2) of combined toxins for 24 h. The cell viability as the main experiment parameter was calculated using this following formula:

Cell Viability (%) = 100 × (Mean OD in sample group − OD in blank)/(Mean OD in control group − OD in blank)(1)

### 5.4. Assessment of the Effect of Mycotoxin Combinations

The types of mycotoxin interactions were assessed according to the combination index (CI)-isobologram equation by the Chou-Talalay method, which is often used for the combined effects of drug combinations. In general, this method plots the median–effect curves of individual and combined mycotoxins from the median-effect principle and equation of the mass-action law:

*fa/fu* = (D/D*m*)^m^ or D = D*m* [*fa*/(1 − *fa*)]^1/m^ (2)

In this formula, D is the value of tested toxin dose, *fa* is the fraction of the cell inhibition rate induced by D, and *fu* is the fraction of cell viability (*fu* = 1 − *fa*). Dm is the median-effect dose (e.g., IC50), and m is the slope of the median–effect curves and signifies the shape of the media-effect plots (m = 1, > 1 and < 1 represent a hyperbolic, a sigmoidal, and a flat sigmoidal shape of concentration–effect curves, respectively). From the above Chou equation, we can obtain a derived formula:

log(*fa*/*fu*) = mlog(D) − mlog(D*m*) (3)

We can plot the median-effect curves from this derived formula. The values of the “combination index” (CI) introduced by Chou indicate the interaction-type of mixtures of two or more tested toxins. More specifically, combination index, CI = 1, CI < 1, and CI > 1 manifest an additive, a synergetic, and an antagonistic effect, respectively. The values of CI was calculated with the Compusyn software package (ComboSyn Inc., Paramus, NJ, USA). The dose reduction index (DRI) represents a multiple of the dose reduction of the dose of tested toxin combinations compared with that of each individual toxin at the same inhibition rate. DRI > 1 and < 1 represent supportable and not supportable dose-reduction; DRI = 1 represents no dose-reduction [26,27,28].

### 5.5. Statistical Analysis

Data were analyzed using two-way ANOVA with a post hoc Bonferroni test for multiple comparisons to assess the differences between groups with Prism software (GraphPad Prism 6, Dr. Harvey Motulsky, San Diego, CA, USA, http://www.graphpad.com/scientific-software/prism/). All probabilities are two-sided, and a value of *p* < 0.001 or *p* < 0.05 was considered statistically significant. Data represents the mean ± SD of three independent experiments. All parameters were calculated through using the Compusyn software package (ComboSyn Inc., Paramus, NJ, USA, http://www.combosyn.com/); for instance, the dose of median-effect (Dm), the slope of the median effect curves (m), the coefficient of linear correlation, the combination index (CI), and reduction index (DRI).

## Figures and Tables

**Table 1:** Dose-effect relationship parameters for the cytotoxicity by individual type B trichothecenes and their mixtures in GES-1 cells.

Mycotoxin	Dose-Effect Parameters			EC_30_	EC_70_
	D*m*	*m*	*r*		
DON	2.99	0.61	0.9814	0.75	11.99
15-ADON	4.77	0.59	0.9904	1.13	20.05
NIV	0.58	0.42	0.9820	0.08	4.36
FX	0.85	0.48	0.9873	0.15	4.97
DON + 15ADON	1.94	0.46	0.9673	0.31	12.24
DON + FX	2.72	0.29	0.9742	0.15	50.51
DON + NIV	0.73	0.41	0.9781	0.09	5.76
15-ADON + FX	4.79	0.40	0.9546	0.58	39.84
15-ADON + NIV	3.23	0.49	0.9757	0.57	18.20
FX + IV	0.18	0.35	0.9557	0.02	2.03

Dm, m, and r represent the median-effect dose (in this case, it is IC50 value), the slope of median-effect curves and the coefficient of linear correlation derived from the experimental data according to the mass-action law. EC_30_ and EC_70_ are the effective concentrations of 30% and 70% of inhibiting cell viability rate.

**Table 2:** Combination index and dose reduction index values for cytotoxicity by individual type B trichothecenes and their mixtures in GES-1 cells.

Mycotoxin	Combination Ratio	10% Cytotoxicity		30% Cytotoxicity		50% Cytotoxicity		70% Cytotoxicity		90% Cytotoxicity	
		CI	DRI	CI	DRI	CI	DRI	CI	DRI	CI	DRI
DON	1:1	0.12	14.59	0.29	5.61	0.52	3.08	0.95	1.69	2.42	0.64
15-ADON			21.29		8.65		4.91		2.79		1.13
DON	3:1	0.06	70.23	0.42	6.51	1.48	1.46	5.47	0.33	47.19	0.03
FX			22.56		3.82		1.25		0.41		0.07
DON	3:1	0.3	33.47	0.39	10.98	0.5	5.45	0.71	2.71	1.5	0.89
NIV			3.8		3.4		4.62		2.95		2.64
15-ADON	3:1	0.73	7.62	1.39	2.6	2.16	1.33	3.43	0.6	7.63	0.23
FX			1.67		0.99		0.71		0.51		0.3
15-ADON	3:1	3.38	4.1	2.29	2.61	1.9	1.97	1.7	1.48	1.69	0.94
NIV			0.32		0.52		0.71		0.98		1.6
FX	1:1	0.07	51.46	0.16	18.3	0.26	9.56	0.43	4.99	1	1.78
NIV			18.18		9.63		6.47		4.34		2.3

CI < 1, =1, and >1 indicates synergistic, additive, and antagonistic effects, respectively. DRI > 1 and < 1 indicate supportable and not supportable dose-reduction; DRI = 1 represents no dose-reduction.
